# Supplementary figures and images for: Molecular modeling and pharmacophore elucidation study of the Classical Swine Fever virus helicase as a promising pharmacological target
Source: PeerJ. 2013 Jun 11;1:e85. doi: 10.7717/peerj.85 (PMC3685396; doi:10.7717/peerj.85)

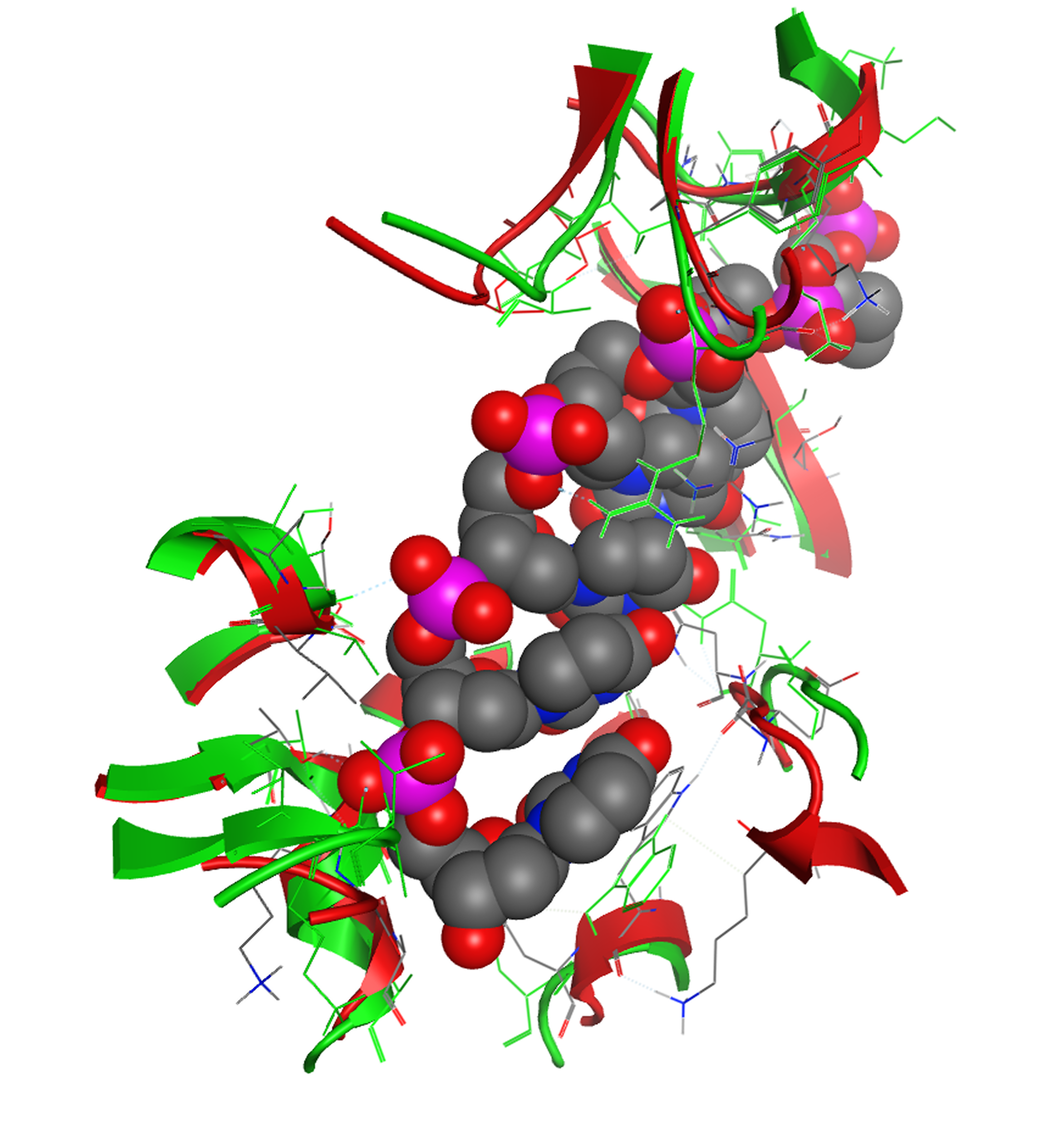

Supplement: Figure S1 — The ssRNA interacting regions are almost identical between the Classical Swine Fever virus helicase model and the Hepatitis C helicase template. [file peerj-01-85-s001.png]

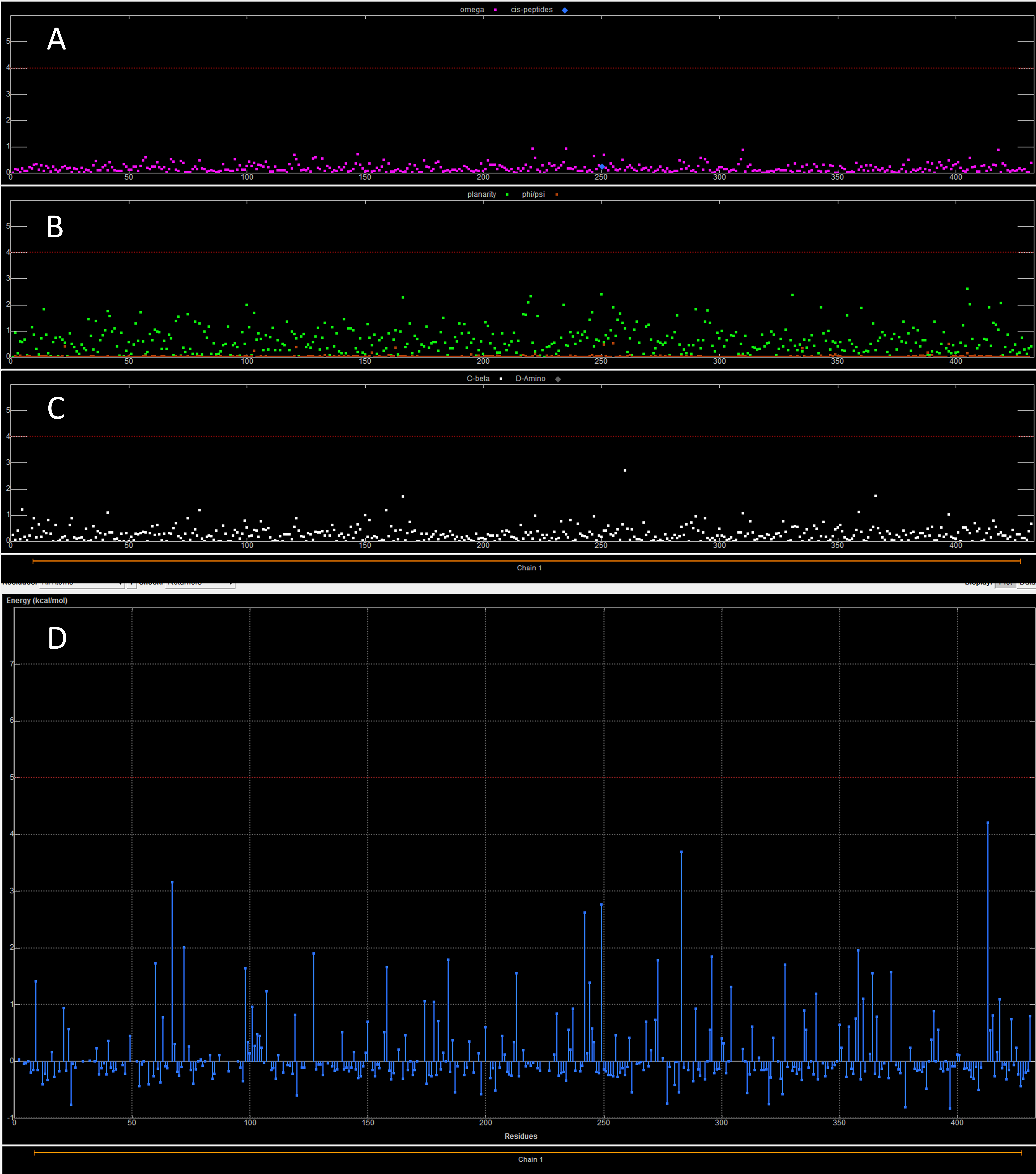

Supplement: Figure S2 — (A) The plot is showing the omega torsion profile. (B) The profiles of phi/psi angles. (C) The planarity the third the C-beta torsion angle profile. (D) the rotamer strain energy profile in kcal/mol. The HCV helicase sequence is on the X-axis. [file peerj-01-85-s002.png]

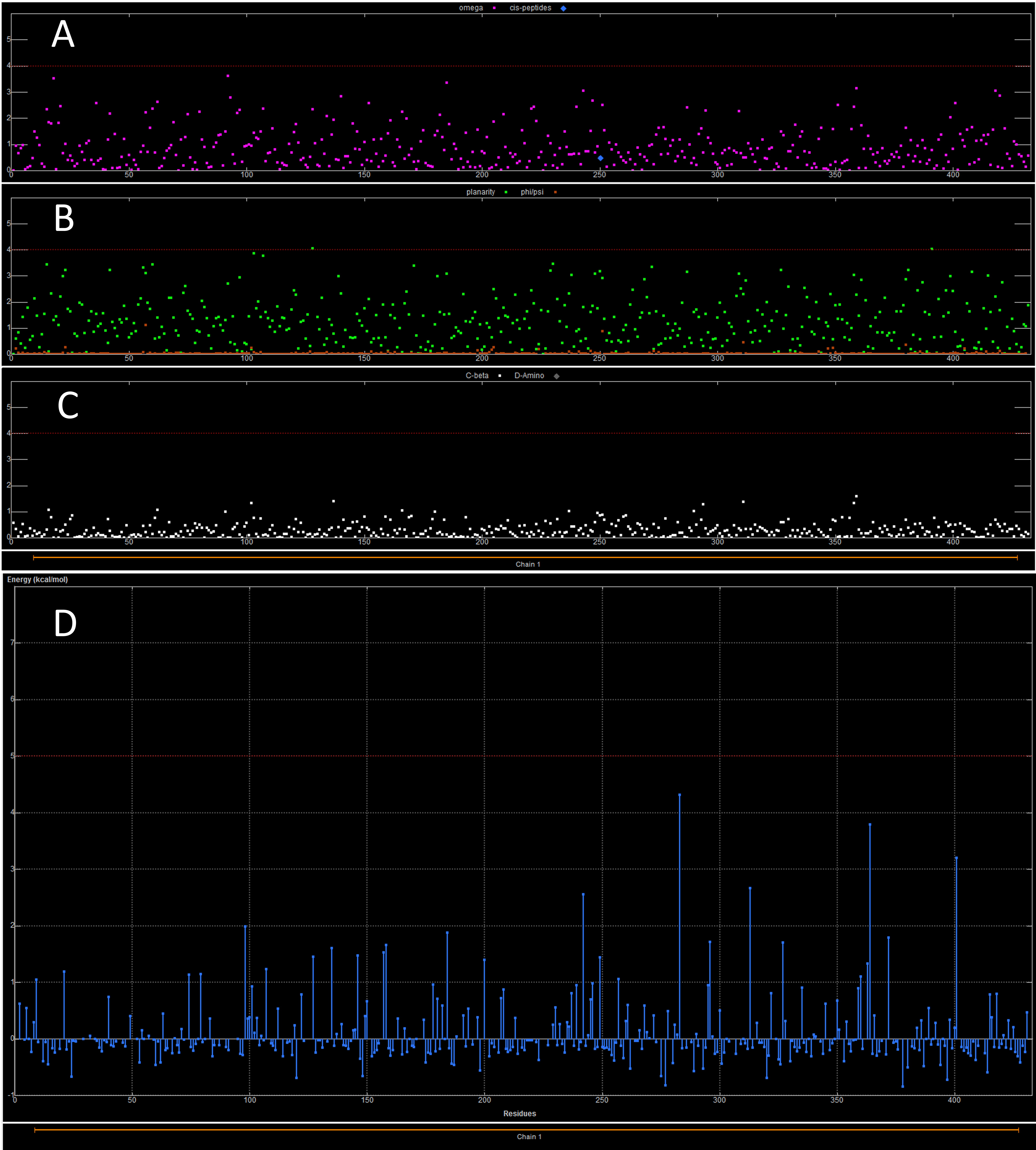

Supplement: Figure S3 — (A) The plot is showing the omega torsion profile. (B) The profiles of phi/psi angles. (C) The planarity the third the C-beta torsion angle profile. (D) the rotamer strain energy profile in kcal/mol. The CSVF helicase sequence is on the X-axis. [file peerj-01-85-s003.png]
